# Association Between Diabetes Medications and the Risk of Parkinson's Disease: A Systematic Review and Meta-Analysis

**DOI:** 10.3389/fneur.2021.678649

**Published:** 2021-07-19

**Authors:** Xiaocui Qin, Xia Zhang, Pinyu Li, Min Wang, Li Yan, Zeqing Bao, Qili Liu

**Affiliations:** ^1^Department of Physiology, Zhaoqing Medical College, Zhaoqing, China; ^2^Department of Pathology and Physiology, Zhaoqing Medical College, Zhaoqing, China; ^3^Department of Pharmacology, Zhaoqing Medical College, Zhaoqing, China

**Keywords:** metformin, dipeptidyl peptidase-4 inhibitors, glucagon-like peptide-1 agonists, glitazones, sulfonylureas

## Abstract

**Background:** Diabetes mellitus (DM) increases the risk of Parkinson's disease (PD). However, whether DM medications play a part on that increased PD risk is unclear. We designed this meta-analysis to assess the influence of different oral DM medications on the PD risk in patients with DM.

**Methods:** We searched PubMed, Embase, and CENTRAL databases for relevant studies up until January 2021. We pooled adjusted outcomes to assess the PD risk in patients using different DM medications including sulfonylurea, metformin, glitazones (GTZ), dipeptidyl peptidase-4 inhibitors (DPP4i), and glucagon-like peptide-1 agonists (GLP1a).

**Results:** We included 10 studies in our analysis. Our results indicate a lack of significant association between the PD risk and the use of sulfonylureas (three studies; HR, 1.26; 95% CI, 0.95 to 1.66; *I*^2^, 70%; *p* = 0.11), DPP4i (three studies; HR, 0.69; 95% CI, 0.35 to 1.38; *I*^2^, 88%; *p* = 0.30), metformin (five studies; HR, 1.23; 95% CI, 0.98 to 1.78; *I*^2^, 84%; *p* = 0.13), and GTZ (six studies; HR, 0.88; 95% CI, 0.66 to 1.16; *I*^2^, 92%; *p* = 0.35). After exclusion of a single study in the GTZ analysis, our results indicate a significantly reduced PD risk with GTZ use (HR, 0.78; 95% CI, 0.65 to 0.93; *I*^2^, 59%; *p* = 0.06). Similarly, after the exclusion of a single study, our results indicate a significantly increased PD risk with the use of metformin (HR, 1.50; 95% CI, 1.11 to 2.02; *I*^2^, 80%; *p* = 0.008). We also found a significantly reduced PD risk with the use of GLP1a (two studies; HR, 0.41; 95% CI, 0.19 to 0.87; *I*^2^, 0%; *p* = 0.02).

**Conclusion:** The role of different DM medications on the PD risk remains unclear, and the quality of studies is low. While our analysis suggests a lack of association between the use of metformin, GTZ, DPP4i, and sulfonylureas and the PD risk, metformin (to a higher degree) and GTZ may still increase the risk. Limited data suggest a protective effect of GLP1a on the PD risk.

## Introduction

Diabetes mellitus (DM) is a metabolic disorder with a high prevalence worldwide. According to statistical data, 493 million people were affected by DM in 2019, and ~700 million people will have the disease in 2045 (1). Despite major advances in therapeutics and management, diabetes-related complications continue to be a major healthcare problem (2). While some complications like nephropathy, retinopathy, and neuropathy are well-recognized, studies suggest that DM increases the risk of neurodegenerative diseases as well (3, 4).

Parkinson's disease (PD) is the second most common neurogenerative disease after Alzheimer's disease (5). The prevalence of PD is higher in the elderly, affecting ~1% of the population above 60 years (6). Lifestyles and genetic risk factors have been implicated in the development of PD (5). However, recent evidence suggests that metabolic disorders like obesity, diabetes, and metabolic syndrome are also independent risk factors for the disease (7). Indeed, a meta-analysis of population-based cohort studies has indicated that DM independently increases the PD risk by approximately 38% (8). The neuroprotective effect of insulin may be the cause of this association (9). Insulin deficiency or resistance leading to a lack of activation of insulin receptors in the brain have been shown to contribute to different neurological disorders including neurodevelopmental syndromes, neoplasms, depression, and neurodegenerative diseases (9). Therefore, whether DM therapies that modulate insulin levels and insulin resistance alter the PD risk needs to be determined (10).

Over the last decade, much research has been conducted on the influence of specific DM drugs and the PD risk in patients with DM. Systematic reviews have pooled the evidence for single drugs like metformin or glitazones (GTZ) based on data from only 3–4 studies each (11–13). The evidence for other DM medications like sulfonylureas, dipeptidyl peptidase-4 inhibitors (DDP4i), and glucagon-like peptide-1 receptor agonists (GLP1a) has not been synthesized. Thus, we conducted a detailed and updated literature search to assess the impact of different oral diabetic medications on the PD risk in patients with DM.

## Materials and Methods

### Inclusion Criteria

We conducted this review as per the PRISMA (Preferred Reporting Items for Systematic Reviews and Meta-analyses) statement (14). However, the review protocol was not registered. The inclusion criteria for the review were the following:

(1) All prospective or retrospective cohort studies conducted on patients with DM. (2) Studies reporting incidence of PD in patients with DM using specific oral DM medications. (3) Studies reporting adjusted or propensity-matched PD incidences in users vs. non-users of a particular diabetic drug (without restrictions on the type of oral DM drug studied).

The following studies were excluded: (1) Studies comparing outcomes between patients with and without DM. (2) Studies failing to report separate data for PD. (3) Studies presenting combined incidences for two or more drugs. (4) Review articles and non-English language studies. For studies presenting data from the same database with the same or overlapping study periods, we only included the study with the largest sample size.

### Search Strategy

Two reviewers independently conducted the electronic search. With the help of a librarian, the databases of PubMed, Embase, and CENTRAL were searched to identify relevant publications. All databases were screened from inception to January 2021. We used the following keywords for the literature search: “Parkinson,” “diabetes mellitus,” “medication,” “risk,” “metformin,” “sulfonylurea,” “glitazones,” “thiazolidinediones,” “glucagon-like peptide,” “dipeptidyl peptidase 4,” “meglitinide,” and “glucosidase inhibitor.” Supplementary Table 1 shows the search strategy. Every search result was evaluated by two reviewers independently (titles and abstracts initially and then full texts). All full texts were reviewed based on the inclusion and exclusion criteria and we only selected articles satisfying all the criteria for this review. Any disagreements were resolved by discussion. To avoid any missed studies, the bibliography of included studies was hand searched for any additional references.

### Data Extraction and Risk of Bias Assessment

We prepared a data extraction form to compile relevant details from the studies. The final version of this template was approved by all the study investigators; it included details of the first author, publication year, study type, location, the database used, study period, sample size, the mean age of the sample, DM medication studied, medication users, factors adjusted for the outcome, outcome data, and follow-ups. Data were extracted independently by two reviewers, and any disagreements were resolved by discussion.

The methodological quality of the studies included was assessed using the Newcastle-Ottawa scale (15). This too was carried out in duplicate and independently by two study investigators. Studies were awarded points for selection of study population, comparability, and outcomes. The maximum possible score was nine. We also assessed the certainty of the evidence using the Grading of Recommendations Assessment, Development, and Evaluation (GRADE) tool using the GRADEpro GDT software [GRADEpro Guideline Development Tool, McMaster University, 2020 (developed by Evidence Prime)].

### Statistical Analysis

We used “Review Manager” (RevMan, version 5.3; Nordic Cochrane Center [Cochrane Collaboration], Copenhagen, Denmark; 2014) for all the meta-analyses. Adjusted hazard ratios (HRs) or related effect sizes of the outcomes were extracted along with the 95% confidence intervals (CIs). Data were pooled using the generic inverse function of the meta-analysis software. A random-effects model was preferred for the meta-analysis. The *I*^2^ statistic was used to assess inter-study heterogeneity. We defined low heterogeneity as *I*^2^ values of 25–50%, medium heterogeneity as values of 50–75%, and substantial heterogeneity as values higher than 75%. Funnel plots were not used to assess publication bias because we included <10 studies per meta-analysis. We conducted a sensitivity analysis or a “leave-one-out” analysis to assess if any study had biased the pooled outcomes. Data of every study was sequentially excluded in the software itself to recalculate the effect size. We performed a sensitivity analysis for meta-analyses with at least three studies. We considered *p* ≤ 0.5 as statistically significant.

## Results

### Search Results and Details of Included Studies

**Figure 1:**
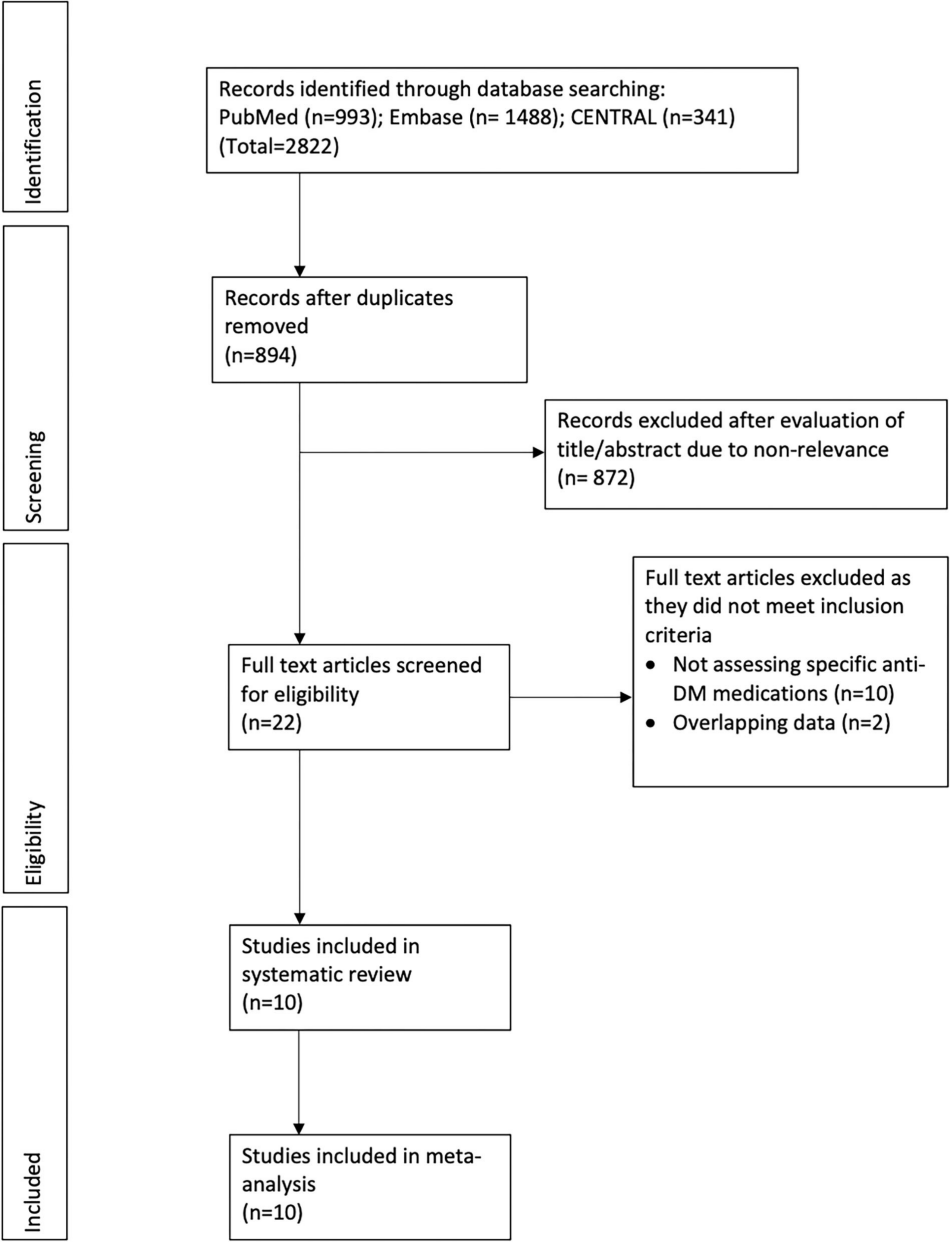
Study flow chart.

The flow chart of the study is presented in Figure 1. We found 894 unique articles after the systematic literature search. Of these, we selected 22 for full-text analysis. Twelve articles were excluded because 10 compared the PD risk between patients with and without DM and did not focus on specific DM medications (16–25) and 2 (26, 27) had overlapping data with another one (28). Finally, 10 studies met the inclusion criteria and were analyzed for this review (28–37). Table 1 presents characteristics of all included studies. All of them were retrospective cohort studies with data extracted from insurance databases or national registries. The sample size varied widely from 5,530 to 1,308,089 patients. The included studies assessed the PD risk among patients with DM using sulfonylureas, metformin, GTZ, DPP4i, GLP1a, meglitinides, or α-glucosidase inhibitors. The studies also varied widely in the factors adjusted for the outcome analysis and the follow-up durations. The Newcastle-Ottawa score ranged between 6 and 8 for the included studies (Table 2).

**Table 1:** Details of included studies.

**References**	**Location**	**Database used**	**Study period**	**Sample size**	**Mean age (Years)**	**DM medication assessed**	**Medication users**	**Adjusted Factors for study outcome**	**Follow-up (years)**
Wahlqvist et al. (29)	Taiwan	Taiwan Health Insurance Database	1996–2007	11,730	64.3 ± 9.6	Metformin Sulfonylurea	1,879 3,431	Date of diagnosis, gender, age, locality, monthly income, level of care, comorbidity index, insulin use	NR
Brauer et al. (30)	UK	United Kingdom Clinical Practice Database	1999–2013	164,970	62.7 (53.9–71.1)^*^	GTZ	7,906	Smoking, alcohol, BMI, head injury, duration of diabetes, glycated hemoglobin, use of calcium channel blockers, hormone replacement therapy	6.1
Connolly et al. (31)	USA	Medicare Database	1997–2005	29,397	77.63 ± 6.88	GTZ Sulfonylurea	5,230 24,167	Propensity score matching	2.97
Svenningsson et al. (34)	Sweden	Swedish Patient Register	2008–2010	5,880	NR	DPP4i GLP1a	67 8	Age, sex, insulin use, education	NR
Brakedal et al. (32)	Norway	Norwegian Prescription Database	2004–2014	102,745	63.45 ± 11.15	GTZ Metformin	8,396 94,349	Age and sex	6.95
Kaun et al. (33)	Taiwan	Taiwan Health Insurance Database	2000–2010	9,302	64.7 ± 9.7	Metformin	4,651	Age, sex, Charlson Comorbidity Index, Adapted Diabetes Complications Severity Index, comorbidities of hypertension, chronic kidney disease, hyperlipidemia, heart failure, arrhythmia, stroke, head injury, and coronary artery disease; and medications of antidiabetes mellitus drug, anti-hypertensive drug, and statin	12
Shi et al. (35)	USA	Veterans Integrated Services Network	2004–2010	5,530	63.24 ± 10.85	Metformin	2,774	Propensity score matching	5.2
Brauer et al. (36)	UK	The Health Improvement Network database	2006–2019	100,288	62.7 ± 12.6	GTZ DPP4i GLP1a	21,175 36,897 10,684	Propensity score matching	2.81–3.6
Rhee et al. (37)	South Korea	National Health insurance Service	2009–2010	1,308,089	60.8 ± 10	Metformin Sulfonylurea GTZ DPP4i Meglitinide α-Glucosidase inhibitor	644,921 710,658 121,375 80,151 42,808 204,867	Age, sex, body mass index, smoking, drinking, and physical activity	6.3
Chang et al. (28)	Taiwan	Taiwan Health Insurance Database	1996–2013	48,828	57.91 ± 10.27	GTZ	24,414	Age, gender, DM duration, Charlson Comorbidity Index, and insulin use	10

* *Median (Interquartile range)*.

*GTZ, glitazones; DPP4i, Dipeptidyl peptidase-4 inhibitors; GLP1a, glucagon-like peptide-1 agonists; DM, diabetes mellitus; NOS, Newcastle-Ottawa Scale*.

**Table 2:** Risk of bias evaluation of individual studies.

**References**	**Selection**				**Comparability**		**Outcome**			**Total**
	**Representative of the exposed cohort**	**Selection of external cohort**	**Ascertainment of exposure**	**Outcome of interest does not present at start**	**Main factor**	**Additional factor**	**Assessment of outcome**	**Sufficient follow-up**	**Adequacy of follow-up**	**(9/9)**
Wahlqvist et al. (29)	+	+	+	+	+	+	+	0	0	7
Brauer et al. (30)	+	+	+	+	0	+	+	+	0	7
Connolly et al. (31)	+	+	+	+	+	+	+	+	0	8
Svenningsson et al. (34)	+	+	+	+	+	+	0	0	0	6
Brakedal et al. (32)	+	+	+	+	+	+	0	+	0	7
Kaun et al. (33)	+	+	+	+	+	+	0	+	0	7
Shi et al. (35)	+	+	+	+	+	+	+	+	0	8
Brauer et al. (36)	+	+	+	+	+	+	+	+	0	8
Rhee et al. (37)	+	+	+	+	+	+	+	+	0	8
Chang et al. (28)	+	+	+	+	+	+	+	+	0	8

### Meta-Analysis

**Figure 2:**
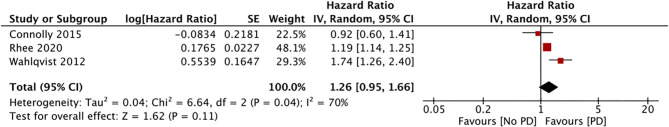
Meta-analysis of PD risk in patients with DM using sulfonylureas.

Only three studies assessed the incidence of PD in patients using sulfonylureas (29, 31, 37). Our pooled analysis of data from 738,256 sulfonylurea users indicate a lack of association between the PD risk and the use of sulfonylureas (HR, 1.26; 95% CI, 0.95, 1.66; *I*^2^, 70%; *p* = 0.11) (Figure 2). The results were stable on our sensitivity analyses without significance changes upon exclusion of any study.

**Figure 3:**
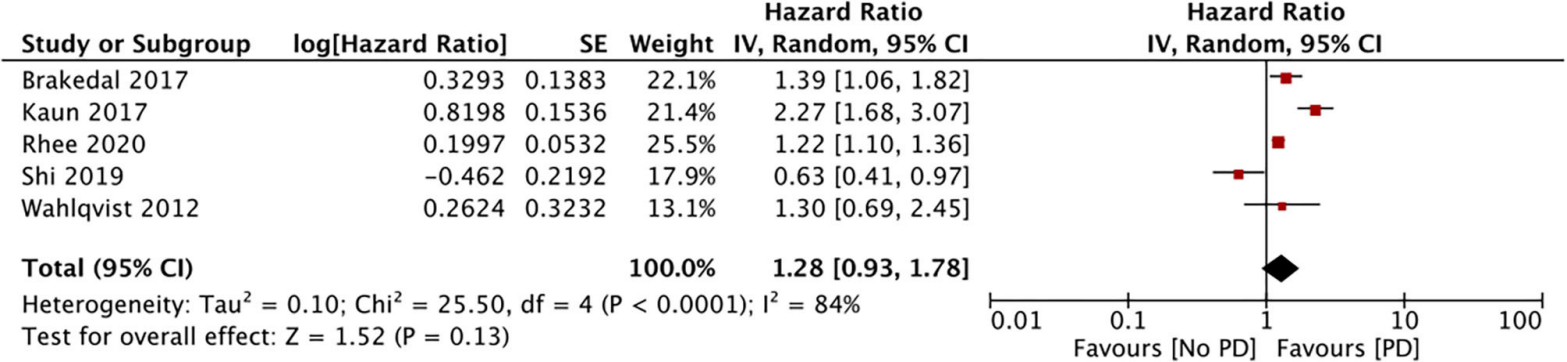
Meta-analysis of PD risk in patients with DM using metformin.

Five studies compared the incidence of PD among metformin users and non-users (29, 32, 33, 35, 37). Meta-analysis results indicate a lack of association between the PD risk and the use of metformin (HR, 1.23; 95% CI, 0.98, 1.78; *I*^2^, 84%; *p* = 0.13) (data from 748,574 metformin users) (Figure 3). After excluding data from the study of Shi et al. (35), the results indicated a significantly increased PD risk for metformin users (HR, 1.50; 95% CI, 1.11, 2.02; *I*^2^, 80%; *p* = 0.008). The results were stable upon exclusion of the remaining studies.

**Figure 4:**
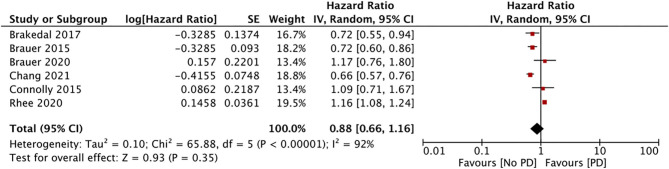
Meta-analysis of PD risk in patients with DM using GTZ.

The impact of GTZ use on the incidence of PD was assessed in six studies (data from 188,496 GTZ users) (28, 30–32, 36, 37). Our pooled analysis failed to demonstrate any significant association between GTZ use and the PD risk (HR, 0.88; 95% CI, 0.66 to 1.16; *I*^2^, 92%; *p* = 0.35) (Figure 4). On the sensitivity analysis, we found the study of Rhee et al. (37) to exert an undue influence on the pooled effect size, and we found a statistically significant reduction in the PD risk after its exclusion (HR, 0.78; 95% CI, 0.65 to 0.93; *I*^2^, 59%; *p* = 0.05). There was no change in the significance of the results on the sequential exclusion of the remaining studies.

**Figure 5:**
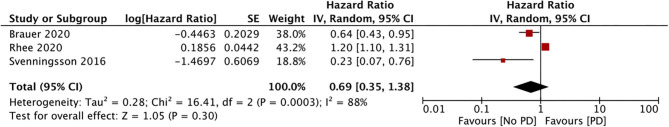
Meta-analysis of PD risk in patients with DM using DPP4i.

**Figure 6:**
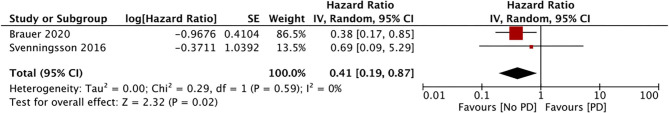
Meta-analysis of PD risk in patients with DM using GLP1a.

Only three studies assessed the incidence of PD with the use of DPP4i (data from 117,115 DPP4i users) (34, 36, 37). Our meta-analysis results showed no association between the use of DPP4i and the PD risk (HR, 0.69; 95% CI, 0.35 to 1.38; *I*^2^, 88%; *p* = 0.30) (Figure 5). The results were stable on sensitivity analyses without significance changes upon exclusion of any study. Finally, just two studies assessed the impact of GLP1a on the PD risk (data from 10,692 GLP1a users) (34, 36). Our pooled analysis results indicate a significantly reduced PD risk for GLP1a users (HR, 0.41; 95% CI, 0.19 to 0.87; *I*^2^, 0%; *p* = 0.02) (Figure 6). The role of meglitinides and α-glucosidase inhibitors on the PD risk was assessed only by Rhee et al. (37). Their results indicated a statistically significant increase in the PD risk for meglitinide users (HR, 1.35; 95% CI, 1.22 to 1.49) and α-glucosidase inhibitor users (HR, 1.26; 95% CI, 1.20 to 1.33). Supplementary Table 2 presents the GRADE evidence assessment. We found the overall quality of the evidence to be very low for all outcomes.

## Discussion

Despite several studies reporting a positive association between DM and the PD risk, the exact mechanism is unclear (8). DM and PD may share pathophysiological processes leading to increased risk of neurodegeneration. The metabolic inflammation associated with DM may have a role in the pathogenesis of PD (38). DM-associated insulin resistance has been implicated in the degeneration of the nigrostriatal dopaminergic pathway (39). Also, PGC-1α, a gene protective for dopaminergic neurons, has been shown to be insufficiently expressed in patients with DM and may contribute to its development (40). Based on this, if anti-diabetic medications that alter insulin levels or insulin resistance have a role in the PD risk, they may provide novel therapeutic opportunities for reducing the incidence or for treatment of PD.

Through our systematic review of the literature, we found that studies have assessed the association between five different classes of oral DM medications (most commonly metformin and GTZ) and the PD risk. The association between metformin use and PD differs according to different studies. Three studies (32, 33, 37) reported an increased PD risk, one (35) reported a reduced risk, and one (29) found a lack of association between metformin use and PD development. Overall, we also found a lack of association between metformin use and PD, but after excluding data from one study (35), the results showed a significant association. In a previous meta-analysis, Ping et al. (11) reported a lack of association between metformin use and the general risk of neurodegenerative diseases, but an increased PD risk in a subgroup analysis of three studies. These paradoxical results between studies can be attributed to inter-study heterogeneity in the populations, drug dosages and duration, classes of drugs, follow-up lengths, and adjusted factors. Also, there may be contradictory effects of a drug during the pathophysiological process of PD. The neuroprotective actions of metformin include a decrease in DM-induced nerve injury (41), an increase in neurogenesis and spatial memory (38), and brain protection from oxidative damage caused by DM (42). On the other hand, prolonged metformin use results in vitamin B12 deficiency, which has been implicated in worsening of PD (43, 44). As vitamin B12 was not adjusted for in any of the studies, it is difficult to derive strong conclusions on the association between metformin use and PD.

GTZ are peroxisome proliferator-activated receptors (PPAR) agonists that improve insulin resistance and reduce hyperglycemia (28). The PPAR pathway is thought to play a major role in the pathogenesis of PD, and GTZ may be an important class of drugs to prevent PD (45). Indeed, animal studies on PD have reported lowering of inflammation, reduced loss of dopaminergic cells, and improvement in motor functions with the use of GTZ (28, 45). Nevertheless, animal studies have inherent drawbacks. A multicentric randomized controlled trial on 210 patients has also evaluated the efficacy of pioglitazone in early PD (46). The study, however, failed to demonstrate any beneficial effect of the drug in patients with PD. On our meta-analysis with the available data, we found no influence of GTZ on the PD risk. However, after the exclusion of the study of Rhee et al. (37), the results indicated a significantly reduced PD risk in GTZ users. The sample size of Rhee et al. (37) was larger than those of all the other studies combined, and their results cannot be easily ignored. Among the remaining studies, three (28, 30, 32) reported a significantly reduced PD risk with GTZ, while Brauer et al. (36) and Conolly et al. (31) found no such association. However, in a sensitivity analysis, Brauer et al. (36) reported a protective effect of GTZ when the follow-up time was censored at the end of GTZ use. On the other hand, the short follow-up period in the study of Conolly et al. (31) may have been an important factor in their non-significant results. A second important variable that could have influenced outcomes is the duration of GTZ exposure. While Rhee et al. (37) failed to describe the duration of GTZ use in their study, Brauer et al. (30) in a sub-group analysis found that GTZ use for more than 3 years (but not short-term use) was protective against PD. Similarly, Chang et al. (28) also reported a beneficial effect of GTZ for long-time users. Thus, although our pooled analysis indicates no association between GTZ use and PD, our results are not confirmatory and additional robust studies are needed.

GLP1a and DPP4i have been used to treat DM since 2005 (34). GLP1a acts on the beta cells of the pancreas and liver, where they stimulate insulin and inhibit glucagon secretion, respectively. The GLP1 receptor is degraded by the enzyme DPP4, and DPP4i indirectly enhances the action of GLP1 by lowering blood glucose levels (47). GLP-1 may attenuate DM-induced neuronal inflammation and enhance insulin signaling in the nervous system by exerting a neuroprotective action (48). Inspired by such potential beneficial effects, two phase 2 trials have evaluated the effect of GLP1a for PD management. In a placebo-controlled trial, Athauda et al. (49) reported a positive effect on off-medication motor scores with exenatide. Moreover, another proof-of-concept study reported clinically relevant improvements in motor and cognitive measures in patients with PD with the same drug (50). However, due to the small size of these trials, a therapeutic role of GLP1a for PD has still not been established. Our own results indicate a significantly reduced PD risk in patients using GLP1a, but no association in patients using DPP4i. These results should be interpreted with caution because only two studies were available for the GLP1a meta-analysis. Additionally, in the study of Svenningsson et al. (34), only eight patients were GLP1a users and, therefore, our results were highly influenced the data from Brauer et al. (36). In the last analysis, we found a lack of association between sulfonylurea use and the PD risk based on only three studies in the analysis.

Our review has strengths and limitations. To enumerate its limitations first, the quality of evidence was low for all outcomes. Also, there was high heterogeneity among the studies included due to methodological differences among them. Foremost, we found significant differences in the confounding factors adjusted for analyses, and this may be a major cause of disparity in their results. Also, the timing and duration of exposure to each particular DM drug varied widely among the patients included in every study, and this may have influenced the PD risk. Moreover, this variable was assessed only in a few of the studies and we could not perform a subgroup analysis. We found no information on the level of diabetes control among the study participants, the nature of other comorbidities, or other concomitant medications taken by them due to the retrospective nature of the studies included. Therefore, those confounding variables may have influenced the study outcomes. In addition, the class of drug against which each DM medication was compared was not homogenous among the studies, and this may have skewed our results. We compared one drug vs. any other drug to assess the PD risk among diabetics, but we cannot tell whether the observed effects were due the study drug or to the compared compound. An ideal situation would have had data comparing a single DM drug with placebo to assess the PD risk in these patients. However, such comparisons are impossible in clinical trials or in real-world scenarios, and the evidence from registries and insurance databases is the only resort to assess such association. Finally, the risk of data entry errors and misclassification of patients cannot be completely ruled out due to the retrospective nature of the studies included. Furthermore, the authors of the two studies from the UK used different databases, but they may have overlapping data (30, 36).

Among the strengths of our study, this is the first comprehensive review assessing the impact of different DM medications on the PD risk. Unlike prior reviews (11–13), ours includes a detailed analysis for every oral DM drug in literature. We obtained data from many new studies and therefore our analysis presents the most updated evidence on the subject. Finally, we conducted sensitivity analyses to assess the stability of our results.

To conclude, the role of different DM medications on the PD risk remains unclear and the quality of the current evidence is low. Our analysis suggests a lack of association between the development of PD and the use of metformin, GTZ, DPP4i, or sulfonylureas, but metformin and GTZ (to a lower extent) may increase the PD risk. Limited data suggest a protective effect of GLP1a against PD. Further research is needed for all DM medications with a focus on the dose and duration of exposure to elucidate the association between DM medications' use and the PD risk.

## Data Availability Statement

The original contributions presented in the study are included in the article/Supplementary Materials, further inquiries can be directed to the corresponding author/s.

## Author Contributions

XQ conceived and designed the study and wrote the paper. XZ and PL were involved in literature search and data collection. MW, LY, and ZB analyzed the data. QL reviewed and edited the manuscript. All authors read and approved the final manuscript.

## Conflict of Interest

The authors declare that the research was conducted in the absence of any commercial or financial relationships that could be construed as a potential conflict of interest.
