# Supplementary material for: Association Between Diabetes Medications and the Risk of Parkinson's Disease: A Systematic Review and Meta-Analysis
Source: Front Neurol. 2021 Jul 19;12:678649. doi: 10.3389/fneur.2021.678649 (PMC8326375; doi:10.3389/fneur.2021.678649)
Supplement: Supplementary file 2 [file Table_2.DOCX]

**Supplementary Table 2: GRADE assessment of certainty of evidence**

| **Certainty assessment** | | | | | | | **Summary of findings** | | | | |
| --- | --- | --- | --- | --- | --- | --- | --- | --- | --- | --- | --- |
| **Participants  (studies) Follow up** | **Risk of bias** | **Inconsistency** | **Indirectness** | **Imprecision** | **Publication bias** | **Overall certainty of evidence** | **Study event rates (%)** | | **Relative effect (95% CI)** | **Anticipated absolute effects** | |
|  |  |  |  |  |  |  | **With comparative group** | **With study drug** |  | **Risk with comparative group** | **Risk difference with study drug** |
| **Sulfonylurea** | | | | | | | | | | | |
| 0 (3 observational studies) | not serious | not serious | serious ^a^ | not serious | none | ⨁◯◯◯ VERY LOW | -/0 | -/0 | **HR 1.26** (0.95 to 1.66) | 0 per 1,000 | **1 fewer per 1,000** (from 2 fewer to 1 fewer) |
| **Metformin** | | | | | | | | | | | |
| 0 (5 observational studies) | not serious | serious ^b^ | serious ^a^ | not serious | none | ⨁◯◯◯ VERY LOW | -/0 | -/0 | **HR 1.28** (0.93 to 1.78) | 0 per 1,000 | **1 fewer per 1,000** (from 2 fewer to 1 fewer) |
| **GTZ** | | | | | | | | | | | |
| 0 (6 observational studies) | not serious | serious ^c^ | serious ^a^ | not serious | none | ⨁◯◯◯ VERY LOW | -/0 | -/0 | **HR 0.88** (0.66 to 1.16) | 0 per 1,000 | **1 fewer per 1,000** (from 1 fewer to 1 fewer) |
| **DPP4i** | | | | | | | | | | | |
| 0 (3 observational studies) | not serious | serious ^c^ | serious ^a^ | not serious | none | ⨁◯◯◯ VERY LOW | -/0 | -/0 | **HR 0.69** (0.35 to 1.38) | 0 per 1,000 | **1 fewer per 1,000** (from 1 fewer to 0 fewer) |
| **GLP1a** | | | | | | | | | | | |
| 0 (2 observational studies) | not serious | not serious | serious ^a^ | serious ^d^ | none | ⨁◯◯◯ VERY LOW | -/0 | -/0 | **HR 0.41** (0.19 to 0.87) | 0 per 1,000 | **0 fewer per 1,000** (from 1 fewer to 0 fewer) |

**CI:** Confidence interval; **HR:** Hazard Ratio

#### Explanations

a. heterogenous comparator group in the included studies

b. Opposing results reported by Shi et al

c. Opposing results reported by Rhee et al

d. Only two studies available with the study of Svenningsson et al of small sample size
